# Therapeutic gas‐releasing nanomedicines with controlled release: Advances and perspectives

**DOI:** 10.1002/EXP.20210181

**Published:** 2022-05-25

**Authors:** Yaw Opoku‐Damoah, Run Zhang, Hang T. Ta, Zhi Ping Xu

**Affiliations:** ^1^ Australian Institute for Bioengineering and Nanotechnology The University of Queensland Brisbane Queensland Australia; ^2^ School of Environment and Science Griffith University Brisbane Queensland Australia; ^3^ Queensland Micro and Nanotechnology Centre Griffith University Brisbane Queensland Australia

**Keywords:** gas‐releasing molecules, gas‐releasing nanomedicines, nanoparticle delivery systems, stimulus‐triggered gas release, therapeutic gases

## Abstract

Nanoparticle‐based drug delivery has become one of the most popular approaches for maximising drug therapeutic potentials. With the notable improvements, a greater challenge hinges on the formulation of gasotransmitters with unique challenges that are not met in liquid and solid active ingredients. Gas molecules upon release from formulations for therapeutic purposes have not really been discussed extensively. Herein, we take a critical look at four key gasotransmitters, that is, carbon monoxide (CO), nitric oxide (NO), hydrogen sulphide (H_2_S) and sulphur dioxide (SO_2_), their possible modification into prodrugs known as gas‐releasing molecules (GRMs), and their release from GRMs. Different nanosystems and their mediatory roles for efficient shuttling, targeting and release of these therapeutic gases are also reviewed extensively. This review thoroughly looks at the diverse ways in which these GRM prodrugs in delivery nanosystems are designed to respond to intrinsic and extrinsic stimuli for sustained release. In this review, we seek to provide a succinct summary for the development of therapeutic gases into potent prodrugs that can be adapted in nanomedicine for potential clinical use.

## Graphical abstract

This review takes a critical look at four key gasotransmitters (CO, NO, H_2_S and SO_2_) with their incorporation into prodrugs known as gas‐releasing molecule. Different nanosystems and their mediatory roles for efficient shuttling, targeting and cleavage of therapeutic gases and the responsiveness to typical internal and external stimuli for gas release from prodrugs have been reviewed extensively.

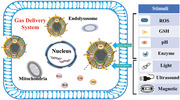

## 1 INTRODUCTION

Advanced drug delivery with nano‐derived systems has already proven to be a reliable way of shuttling drugs to remote diseased sites in laboratory and clinical studies.^[^ ^1^ ^]^ Their ability to control the release of drugs within tumour tissues, inflammatory and cardiovascular sites as well as cells is well documented. The enhanced permeation and retention (EPR) effect, as the primary delivery strategy for tumour therapy, has been a pivotal aspect in nanodrug delivery.^[^ ^2^ ^]^ The ability to intelligently engineer nanodrugs of different shapes and sizes is a magical dimension that enhances their shuttle to targeted sites and their uptake by cells. This technique has been employed to deliver different therapeutic agents for therapy and diagnosis in a variety of diseases.^[^ ^3^ ^]^ It is not surprising that nanomedicines stand tall among diverse techniques to help deliver gaseous therapeutic agents without triggering undesirable immune responses and unwanted gas leakage during circulation that may result in serious side effects.^[^ ^4^ ^]^

Therapeutic gases are shapeless and compressible molecules with weak intermolecular forces and high kinetic energy employed to manage or cure ailments.^[^ ^5^ ^]^ Gasotransmitters are specific gases generated in the body and are capable of mediating various signalling pathways. The production of these gasotransmitters is very common in human and animal bodies where they are employed as biological messengers with medicinal characteristics for treatment of cancers, gastrointestinal and cardiovascular disorders.^[^ ^6^ ^]^ Nitric oxide (NO), oxygen (O_2_), nitrous oxide (N_2_O), carbon monoxide (CO), methane (CH_4_), carbon dioxide (CO_2_), hydrogen sulphide (H_2_S), sulphur dioxide (SO_2_), hydrogen cyanide (HCN), hydrogen (H_2_), ammonia (NH_3_) and ethylene (CH_2_CH_2_) are known examples of gases for therapeutic purposes. The most notable gasotransmitters with well‐documented drug‐like properties are CO, NO, H_2_S and SO_2_.^[^ ^7^ ^]^ All these gases have their respective therapeutic and toxic levels in the human body (<100 ppm for most gasotransmitters).^[^ ^8^ ^]^ The main constraint is the difficulty in directly delivering these therapeutic gases for medical use. The advent of nanotechnology for drug delivery may provide a way to develop gas‐releasing nanomedicines.^[^ ^9^ ^]^

**FIGURE 1:**
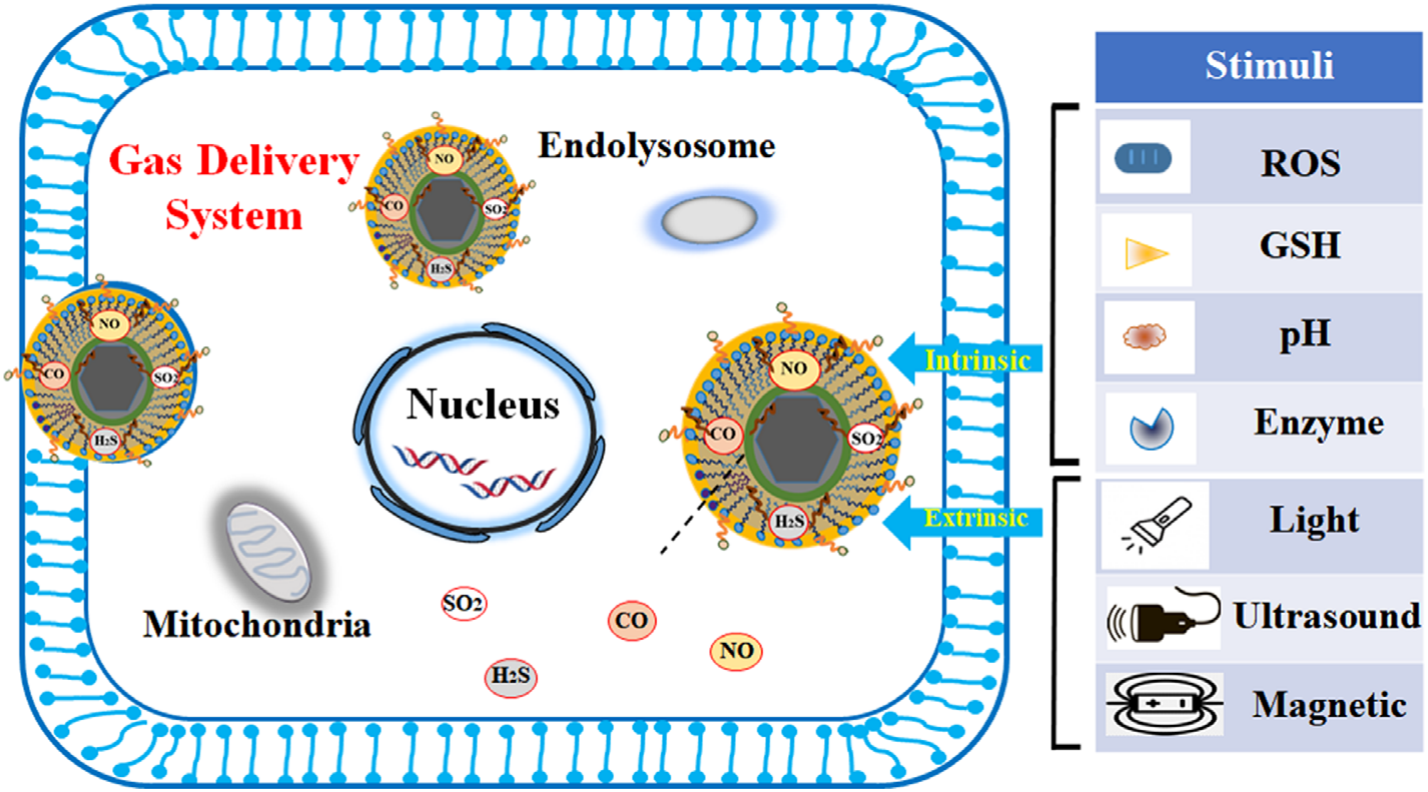
Schematic representation of in situ gas release from the nanoplatform containing GRMs via an intrinsic or extrinsic stimulus applied

In recent years, there have been several innovative strategies to incorporate therapeutic gas‐releasing molecules (GRMs) (prodrugs) into delivery systems.^[^ ^10^ ^]^ One major development is that these prodrugs release gases under certain conditions or triggers. Prodrugs are compounds with relatively low to no therapeutic activity, and can be successfully stimulated to release the active gas by intrinsic (e.g., enzymatic/chemical reactions), extrinsic stimuli (e.g. light/ultrasound/magnetism) or their combination.^[^ ^11^ ^]^ Prodrugs are typically ideal for gases such as NO, CO, SO_2_ and H_2_S since prodrug molecules can be readily loaded and delivered in the nanoparticle form, and then transferred to the active gas under a stimulus or trigger. This property has brought a lot of attention to develop GRMs such as NO‐releasing molecules (NORMs) and CO‐releasing molecules (CORMs). These gas releasing molecules have been explored for several years with continuous improvements in their solubility, bioavailability and suitability as potent prodrugs.^[^ ^12^ ^]^ The active gas is normally conjugated to a molecular frame as a prodrug, which is consequently incorporated into the delivery nanosystem to harness its capability for targeted cellular delivery, efficient release, metabolism and excretion. In particular, the prodrug is cleaved by a required stimulus to release the gas molecule in cells for action, as schematically shown in Figure 1.

**FIGURE 2:**
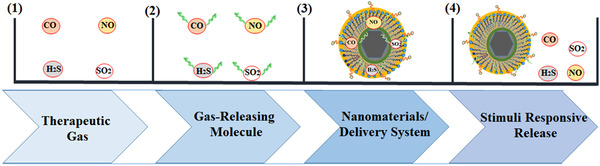
Typical gas delivery strategy starting from development of GRMs (step 2), to loading them into nanomaterial delivery systems (step 3) and stimulus‐responsive release of therapeutic gases (step 4)

In this review, we first briefly discuss the basic biological functions and therapeutic activity of these four gases and responsive versatile GRMs that intelligently harness intrinsic and extrinsic stimuli for local gas release. We then focus on different types of nanosystems that are explored as novel platforms for encapsulating, mediating and delivering therapeutic gas‐release molecules for controlled release, as schematically described in Figure 2 (step 3 and 4). Finally, we compare the advantages and disadvantages of these stimuli and delivery nanosystems, and provide the prospects for future investigations. As far as we know, previous publications have not thoroughly discussed nano‐based systems for delivery of these major gasotransmitters, and thus this review aims to assist researchers by providing a concise set of scientific information for development of nanoparticle‐based gas prodrug delivery platforms.

## 2 THERAPEUTIC POTENTIAL OF GASOTRANSMITTERS AND THEIR TYPICAL PRODRUGS

In 1993, it was established that some gasotransmitters could be produced in the human body for specific biological roles.^[^ ^13^ ^]^ This has led to a lot of investigations to uncover their roles and any possible potentials for gas therapy (GT).^[^ ^14^ ^]^ This section briefly reviews the therapeutic potentials of the most‐studied gasotransmitters, including NO, CO, H_2_S and SO_2_, and their GRMs recently developed. As summarised in Table 1, the therapeutic effects of these most popular gasotransmitters are highlighted together with their drug‐like properties.

**TABLE 1:** Summary of therapeutic effects of four gasotransmitters

Gasotransmitter	Therapeutic effects	Ref.
NO	Production of RNS, such as peroxynitrite Overproduction of ROS Deamination of DNA base Impediment of cellular bioenergetics Downregulation of drug efflux‐related P‐glycoproteins	[16]
CO	Production of anti‐Warburg effect Promotion of tumour bioenergetics Instigation of metabolic exhaustion Induction of oxidative stress Inhibition of mitochondrial respiratory metabolism	[17]
H_2_S	Inhibition of mitochondrial cytochrome‐c oxidase Regulation of mitochondrial function Inhibition of mitochondrial phosphodiesterase.	[18]
SO_2_	Consumption of excess GSH Induction of protein oxidative damage Induction of oxidative stress in tumour cells	[19]

### 2.1 NO

NO is naturally involved in a variety of biological processes, which are now well documented and exploited to enable NO therapy to become a reality. NO is synthesised by an enzymatic reaction involving NO synthase (NOS) and L‐arginine and nicotinamide adenine dinucleotide phosphate (NADPH).^[^ ^15^ ^]^ A term, that is, nitrate‐nitrite‐NO pathway, has been specifically given to NO generation by the sequential reduction of nitrate found in food.

Early researchers have discovered that the endothelium‐derived relaxation factor (EDRF) is NO gas.^[^ ^20^ ^]^ Inhaled NO has been applied in various treatments of several diseases, such as cancers and cardiovascular diseases.^[^ ^21^ ^]^ In fact, all the three forms of NOS, including neuronal NOS, endothelial NOS and inducible NOS have been detected in different kinds of tumours. The concentration of NO in tumour tissues directs its effect on cancer cells. A higher concentration results in DNA‐base deamination, cellular function impairment and enzyme nitrosylation, while a lower concentration facilitates tumour progression.^[^ ^22^ ^]^ NO is capable of circumventing drug resistance in multidrug resistant malignancies through processes including stimulation of blood vessels around cancerous tissues and interference with the dysregulated pro‐survival/anti‐apoptotic NF‐κΒ/Snail/YY1/RKIP/PTEN loop.^[^ ^23^ ^]^ The antibacterial action, wound healing effect and the effect on cardiovascular conditions are well known in scientific research.^[^ ^24^ ^]^

**FIGURE 3:**
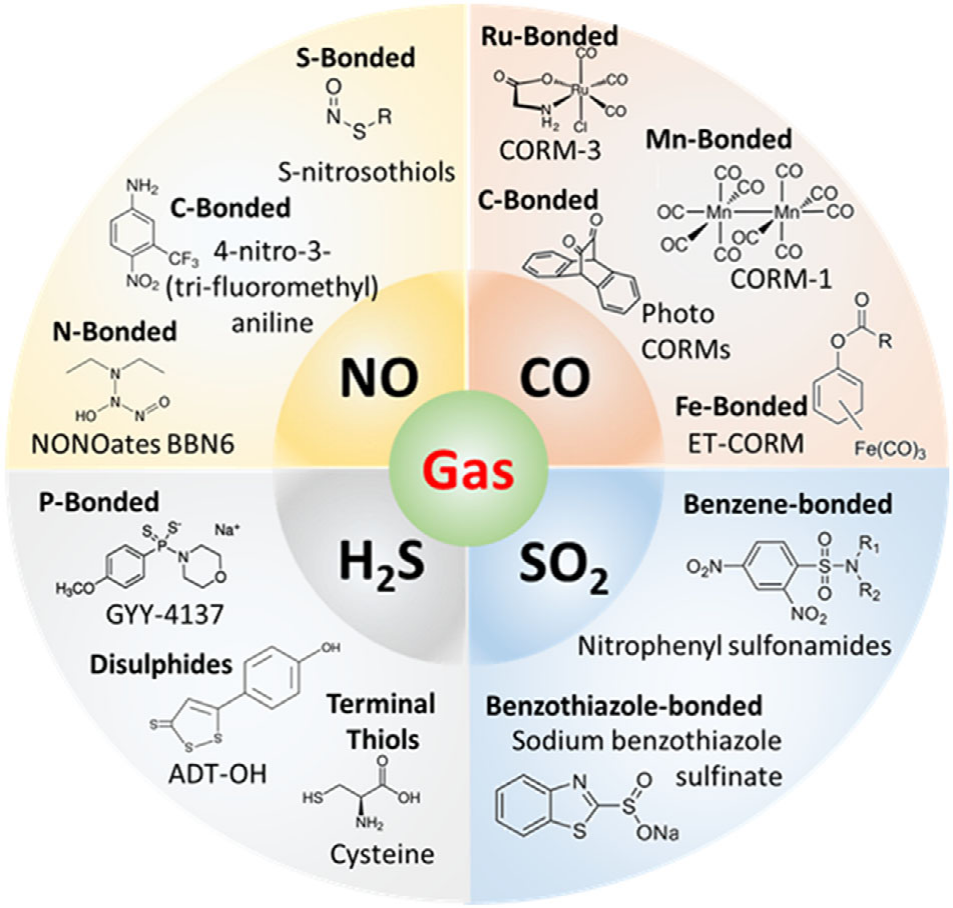
Typical examples of GRMs classified on the basis of specific bonds that are used for their conjugation/bonding and broken for gas release

Recently, researchers have become aware of NO's roles in the biological system and the NO‐releasing mechanism of prodrugs for treatment. For instance, one of the most popular prodrugs for angina known as glyceryl trinitrate releases NO gas through enzymatic reaction.^[^ ^25^ ^]^ Compounds such as S‐nitrosothiols, NONOates and NO adducts, are further developed as NO‐releasing molecules for treatment of various diseases.^[^ ^26^ ^]^ The major challenge is the site‐specificity and precision related to NO release at diseased sites instead of healthy organs. The advent of nitrosyl complexes has changed the narrative and has directed a research path to produce viable pharmaceutical NO‐releasing prodrugs for potential clinical use (Figure 3).

### 2.2 CO

CO is a known toxic gas, which is highly lethal at doses around 1% (10,000 ppm). It is able to displace systemic oxygen bound to haemoglobin of red blood cells, resulting in toxicity.^[^ ^27^ ^]^ Biologically, CO is produced as a result of the enzymatic activity of heme oxygenase (HO), degrading heme to produce iron, biliverdin and CO.^[^ ^28^ ^]^ However, this gas has emerged as a therapeutic agent for effective cancer therapy.

CO possesses anti‐inflammatory properties through a mechanism that involves the mitogen‐activated protein kinases. CO is involved in down‐regulation of pro‐inflammatory proteins such as IL‐1β/TNF‐α.^[^ ^29^ ^]^ In some cancers, HO‐1 is localised in cells, and CO produces an anti‐Warburg effect by quickly aiding tumour bioenergetics, which may end up in metabolic exhaustion. CO also targets mitochondria in tumour cells to trigger the generation of excess ROS, resulting in mitochondrial collapse in cancer therapy.^[^ ^30^ ^]^ The expression of HO‐1 by endothelial cells and the subsequent production of CO could prevent platelet aggregation and inhibit apoptosis in endothelial cells.^[^ ^31^ ^]^ For instance, Sato et al. confirmed that the protective effect of HO‐1 in mouse‐to‐rat cardiac transplant is directly related to the generation of CO, which inhibits platelet aggregation, the cause of vascular thrombosis and myocardial infarction.^[^ ^32^ ^]^ CO is also capable of activating soluble guanylyl cyclase (sGC) and producing cGMP through a corresponding reaction with its heme moiety. cGMP eventually plays the role of a second messenger for diverse cellular function signals, leading to vasodilation and inhibiting platelet aggregation.^[^ ^33^ ^]^

Previously, CO gas was delivered through the lung in a very risky process. It has been reported that the carboxyhemoglobin level in the body can reach about 20% upon exposure to 1000 ppm CO for 30–60 min.^[^ ^34^ ^]^ There have also been attempts to use microbubbles and liposomes to carry and deliver CO gas directly with the aid of acoustically activated ultrasound stimuli.^[^ ^35^ ^]^ In the early 2000s, CORMs were eventually introduced by Motterlini et al. who designed novel pharmaceutical prodrugs that hold CO from leaking until the prodrugs reach the specified target and then release CO upon excitation by an intrinsic or extrinsic stimulus.^[^ ^36^ ^]^ As shown in Figure 3, CO gas molecules are attached to a functional group in a stable manner before they are released upon triggering by a carefully selected stimulus. This has shaped the way that CO is administered in recent years, by developing a few new generations of CORMs for efficient drug delivery and good biocompatibility.^[^ ^37^ ^]^ New generation of nano‐based CO‐prodrug delivery systems is thus becoming a promising way to utilise CORMs for improved delivery and bioavailability.

### 2.3 H_2_S

H_2_S is another important gasotransmitter studied in recent years. It is produced by L‑cysteine and catalysed by two key enzymes in the human body known as cystathionine γ‐lyase and cystathionine β‐synthase.^[^ ^38^ ^]^ H_2_S is a novel signalling molecule in some cellular functions as a regulator of apoptosis, perfusion and inflammation.^[^ ^39^ ^]^ The inhibitory effect of H_2_S on mitochondrial cytochrome‐c oxidase is well characterised, and this is generally assumed to be the main cause of H_2_S toxicity. Researchers have already established that H_2_S‐synthesising enzymes are upregulated in various cancer cells.^[^ ^40^ ^]^ The characteristic H_2_S over‐production has been suggested to modulate cancer cell adaptation via persulphidation of key proteins in various signalling pathways.^[^ ^41^ ^]^ While this over‐expression leads to a pro‐cancer ability, the exogenous addition of H_2_S can produce an anti‐cancer effect, depending on the level and exposure time,^[^ ^42^ ^]^ which has raised investigations towards H_2_S‐releasing molecules for possible therapy purposes.

Novel H_2_S‐releasing molecules have been designed and examined to produce this gas in biological systems.^[^ ^43^ ^]^ The most common strategy is to replace an oxygen atom in a molecule with sulphur in order to generate H_2_S upon hydrolysis. Another common approach is to design molecules that generate an intermediate persulphide, which is subsequently cleaved by thiols to generate H_2_S and a disulphide (Figure 3). A more advanced technique is to develop new molecules that release carbonyl sulphide (COS) as an intermediate, which can be then converted rapidly to H_2_S by the ubiquitous mammalian enzyme carbonic anhydrase.^[^ ^44^ ^]^

### 2.4 SO_2_

SO_2_ fulfils the criteria for gasotransmitter classification and is also endogenously produced from sulphur‐containing amino acids in biological systems. SO_2_ and pyruvate are products of L‐cysteine oxidisation by cysteine dioxygenase to L‐cysteine sulphinate and then to β‐sulphinylpyruvate.^[^ ^45^ ^]^ SO_2_ can also be synthesised from H_2_S oxidation. This gas is toxic at high levels when inhaled, and causes oxidative stress‐induced damage of proteins, lipids and DNAs.^[^ ^46^ ^]^ Therapeutic roles of SO_2_ in vasorelaxation, anti‐inflammatory and antibacterial treatment in biological systems have also been reported in recent years.

There are a few methods used to prepare SO_2_‐releasing molecules for therapeutic purposes. These include mixed sulphite salts (NaHSO_3_ and Na_2_SO_3_), overexpression of two isoenzymes of aspartate aminotransferase‐AAT (an upstream enzyme in the SO_2_ generation pathway that catalyses the transamination of L‐cysteine sulphinate), benzosultine, 2, 4‐dinitrophenylsulphonamides, benzosulphones, sodium benzothiazole sulphinates and esterase‐sensitive cyclopropyl esters connected to sulphonate moieties^[^ ^47^ ^]^ (Figure 3). The interest in this gas has increased tremendously over the last years, with many research groups producing SO_2_‐releasing molecules, which can be loaded to nano‐based delivery systems for targeted release and therapy for various diseases.

## 3 MEDIATORY ROLES OF NANOMATERIALS FOR EFFECTIVE GAS DELIVERY AND RELEASE

The advent of GRMs has also paved the way for nano‐inspired delivery materials to help shuttle and release these gas molecules to specific targets. Nanotechnology as a pivotal aspect of drug delivery has proven to make drug uptake more efficient. Nanoparticle‐based systems enable highly responsive, spatial, temporal and dosage‐controlled delivery. Therefore, an organised documentation on the novel nano‐systems and their specified mediatory roles in controlling gas release are important for researchers to design new responsive gas‐release nanomedicines. As seen in Figure 2, the strategy may include conversion of gases to GRMs, encapsulation of GRMs within nanoparticle‐based systems and gas releasing in a controlled pattern within the required biological tissue upon exposure to intrinsic or extrinsic stimuli. In this section, we succinctly discuss the various nanomaterials that have proven novel in encapsulating, successfully delivering, modulating and triggering the release of therapeutic gases.

### 3.1 Inorganic nanomaterials

Biofriendly inorganic materials have been extensively explored for drug loading and delivery. These nanomaterials can be explained in detail to define their roles related to the interesting nature of gases and the responsive release. Some metal and metal‐oxide nanoparticles are sensitive to intrinsic and extrinsic stimuli, and used as photosensitisers or magneto‐responsive materials to orchestrate gas release. Some of the novel and efficient inorganic nanomaterial delivery systems have been discussed herein.

#### 3.1.1 Photothermal agents

Photothermal agents are one of the most frequently used nanomaterials for many delivery systems that require conversion of light to heat energy.^[^ ^48^ ^]^ Currently, six types of NIR laser‐driven photothermal agents are commonly used, including gold (Au) nanostructures (such as Au nanorods and nanoshells),^[^ ^49^ ^]^ carbon‐based materials (such as carbon nanotubes and graphenes),^[^ ^50^ ^]^ copper‐based materials,^[^ ^51^ ^]^ iron oxide materials, synthetic dyes and other synthetic compounds based on the NIR absorption‐to‐thermal conversion mechanism.^[^ ^52^ ^]^ In gas delivery, heat is essential for various bond cleavage and photothermal agents are often utilised together with gas release molecules in nano‐based delivery systems.

**FIGURE 4:**
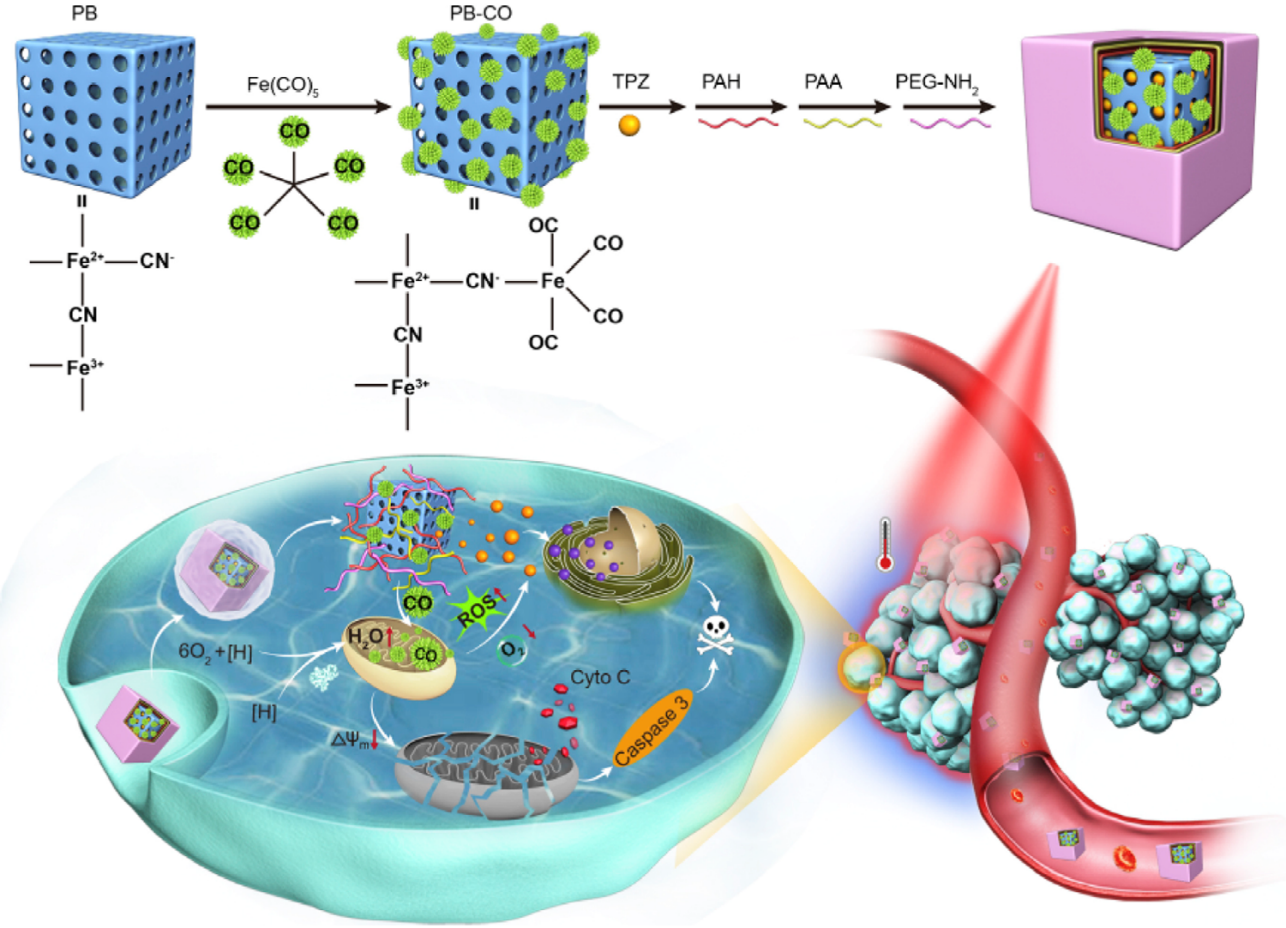
Schematic illustration of polymer‐encapsulated PB‐CO‐TPZ nanoparticles with enhanced bioreductive chemotherapy and CO‐mediated pro‐apoptotic GT upon NIR light irradiation. Adapted with permission.^[^ ^54^ ^]^ Copyright 2019, Elsevier

For instance, mesoporous Prussian blue nanoparticles (PB NPs) were used in many studies to help elevate temperatures to trigger the release of gases in a responsive manner.^[^ ^52^, ^53^ ^]^ Yin et al. constructed an iron‐based FDA‐approved material into nanoparticles for CORM delivery and CO release.^[^ ^54^ ^]^ They incorporated pentacarbonyl iron (Fe(CO)_5_) (as CORMs) to PB nanoparticles via coordination and tirapazamine (TPZ) into the mesopores. As seen in Figure 4, NIR light irradiation increased the tumour temperature to above 50°C and this less‐invasive photothermal effect of PB NPs released CO, which accelerated mitochondrial oxygen consumption and generated hypoxia to activate TPZ.^[^ ^54^ ^]^ Interestingly, the PB nanoparticles also helped overcome multidrug resistance via CO‐induced metabolic exhaustion in the presence of DOX.

NIR light‐triggered photothermal H_2_S‐release nanoplatform was developed by Li et al. for synchronous photothermal effect and gas release to induce cancer cell apoptosis. Polyethyleneimine‐dithiocarbamate was employed as H_2_S prodrug and assembled on a photothermal agent (reduced graphene oxide (rGO) nanosheet). rGO nanosheets converted the NIR laser at 780 nm into thermal energy and elevated the temperature to above 45°C, which subsequently induced H_2_S release and cell apoptosis.^[^ ^55^ ^]^ GO NIR light‐responsive nanovehicles loaded with *bis*‐*N*‐nitroso compounds (BNN6, NORM) were explored in a methacrylate‐modified gelatin (GelMA)/hyaluronic acid graft dopamine (HA‐DA) hydrogel.^[^ ^56^ ^]^ Bismuth sulphide (Bi_2_S_3_) nanoparticles and *N*,*N*′‐Di‐sec‐butyl‐*N*,*N*′‐dinitroso‐1,4‐phenylenediamine (BNN) were also employed for NO‐enhanced mild photothermal therapy upon 808 nm irradiation.^[^ ^57^ ^]^

#### 3.1.2 Photon‐to‐photon conversion agents

Photon‐to‐photon conversion agents such as lanthanide upconversion nanoparticles (UCNPs) have been engineered to absorb NIR lights to emit UV–vis photons.^[^ ^58^ ^]^ Such an idea is very much suitable for triggering gas release because gas release molecules are more sensitive to light in the UV–vis region than in NIR region.^[^ ^59^ ^]^ Moreover, NIR light has deeper penetration into tissues, allowing UV–vis lights to be generated in situ for efficient gas release. The biocompatibility and less toxic nature of these nanoparticles to healthy tissues make them attractive for GRM delivery and on‐demand release. For instance, Li et al. designed a core‐shell‐structured NaYF_4_:Tb,Tm@NaYF_4_ UCNPs encapsulated with a layer of SiO_2_ and covalently linked to a potent NO‐releasing molecule (S‐nitroso‐N‐acetyl‐dl‐penicillamine/SNAP).^[^ ^60^ ^]^ Under 980 nm light irradiation, the UCNP system converted NIR to UV, which broke the chemical bond in SNAP molecule and triggered NO release. In another study, Ou et al. designed a luminescence‐enhanced nanoplatform by doping Ca^2+^ into NaYF_4_:Yb^3+^/Tm^3+^ UCNPs and coating with the NaGdF_4_ shell. These UCNPs were further modified with methyl‐β‐cyclodextrin (M‐β‐CD) and loaded with ruthenium nitrosyl complexes [(3) Ru(NO)(Cl)] as NORMs. Under 980 nm light irradiation, NO was released efficiently.^[^ ^61^ ^]^ Recently, Opoku‐Damoah et al. constructed lanthanide‐based UCNPs by coating lipids on the surface and loading CORMs as a core‐shell structure. They demonstrated the release of CO gas molecules by converting 980 or 808 nm lights to 360 nm UV to efficiently break the specifical bond in CORMs in the presence or absence of other chemotherapeutic agents.^[^ ^62^ ^]^

#### 3.1.3 Catalytic metal ionic species

Gas release via catalytic reactions initiated by metal ionic species is particularly popular for NO, and to some extent, CO therapy.^[^ ^63^ ^]^ In particular, NO releasing from *S*‐nitrosothiols has been observed in the presence of Cu(II), Pd(II), Au(III), Pt(II) and V(III) in the nanosystems. Co(II), Fe(II), Fe(III), Mn(II), Ni(II), Ag(I)^+^ and Sn(II) have also been explored in some situations.^[^ ^64^ ^]^ Dicks et al. proposed a possible general mechanism for these types of catalytic metal ionic species. For example, Cu(I) is possibly produced through reduction of Cu(II) by thiolate anion and formation of RS‐Cu^+^ intermediate in the first step. Cu(I) is then bound to the nitrogen atom and subsequently releases NO.^[^ ^65^ ^]^

Major et al. demonstrated that an NO generator based on Cu(0)‐nanoparticle (80 nm) coated with hydrophilic polyurethane (SP‐60D‐60) combined with the intravenous infusion of RSNO, *S‐*nitroso‐*N*‐acetylpenicillamine (SNAP), released NO in rats.^[^ ^66^ ^]^ Kulyk et al. demonstrated an approach in which copper(II) oxide‐silica catalytically decomposed endogenous NO‐bearing metabolites to generate NO. They confirmed that Cu(II) is reduced to Cu(I) by endogenous thiols present in the blood or in nanomaterials, and Cu(I) reacts with S‐nitrosoglutathione (GSNO) to liberate NO by the subsequent transfer of an electron to form a thiolate anion, which is eventually protonated to produce a thiol and regenerate Cu(II).^[^ ^67^ ^]^

#### 3.1.4 Photocatalytic nanomaterials

**FIGURE 5:**
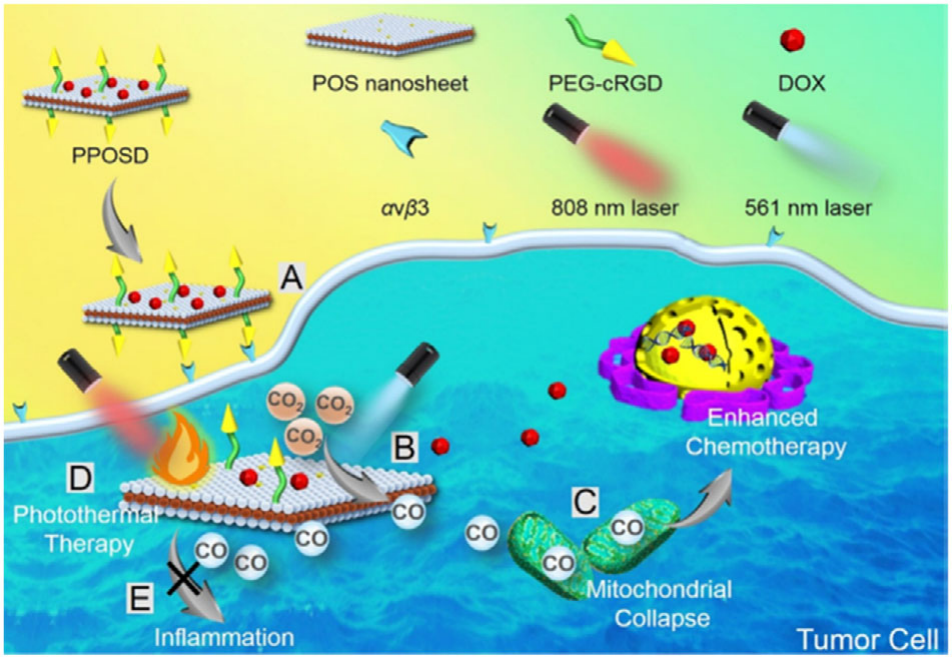
Schematic illustration of PPOSD for enhanced tumour inhibition and anti‐inflammation. Adapted with permission.^[^ ^70^ ^]^ Copyright 2019, American Chemical Society

Gas releasing nanoplatforms with photocatalysts are excited by light absorption to generate electron‐hole pairs in inorganic materials and the holes are separated and transferred to different sites for redox reactions. An interesting research is reduction of CO_2_ for CO therapy. The effective strategy to improve CO_2_ conversion to CO is to increase the CO_2_ adsorption ability of photocatalysts, as electron transfer from the catalytically active site to CO_2_ largely relies on intimate and stable binding interactions with CO_2_ molecule.^[^ ^68^ ^]^ In a quest to optimise the conversion rate of CO_2_ to CO, a photocatalytic nanomaterial (HisAgCCN) was constructed to transform CO_2_ to CO gas for cancer therapy.^[^ ^69^ ^]^ This metal‐organic framework (MOFs)‐based photocatalytic material was designed by modifying Ag_3_PO_4_‐doped carbon‐dot‐decorated C_3_N_4_ nanoparticles (AgCCN) with histidine‐rich peptides. Under 630 nm light irradiation, the nanomaterial photo‐reduced intrinsic CO_2_ to CO for GT.^[^ ^69^ ^]^ In a similar study, Wang et al. prepared partially oxidised tin disulphide (SnS_2_) nanosheets (POS NSs) and incorporated DOX into the nanosystem.^[^ ^70^ ^]^ As described in Figure 5, the polymer@POS@DOX (PPOSD) nanosheets selectively accumulated in tumour tissues via the cRGD‐mediated tumour recognition. Upon 561 nm laser irradiation, the POS moiety in PPOSD photo‐reduced CO_2_ to CO molecules, which subsequently sensitised the chemotherapeutic effect of DOX. The POS in PPOSD also acted as an effective photothermal therapy (PTT) agent upon 808 nm laser irradiation,^[^ ^70^ ^]^ coordinating the tumour cell apoptosis.

Unlike other gas‐releasing nanomedicines, loading GRMs is not required for photocatalytic nanomaterials, while the development of this in situ gas generation system is still in an early stage with limited availability of materials. More efficient nanomaterials are needed to produce a high photocatalytic yield for efficient therapy and the amount of various precursors at diseased sites is a key factor for this technique.

### 3.2 Polymeric nanomaterials

Polymer‐based nanomaterials have been extensively used in cancer drug delivery and they are able to load different drugs through conjugation and encapsulation into the polymer matrix.^[^ ^71^ ^]^ The main types of polymers include amphiphilic core‐shell polymers (polymeric micelles), hydrogels and hyperbranched macromolecules (dendrimers).^[^ ^72^ ^]^ Both synthetic (e.g. poly(ethylene glycol) (PEG), poly(lactic acid) (PLA), poly(ε‐caprolactone) (PCL), poly(lactic‐*co*‐glycolic acid) (PLGA) and *N*‐(2‐hydroxypropyl)‐methacrylamide copolymer (HPMA)) and naturally existing polymers (e.g. albumin, gelatin, chitosan, dextran and heparin) have been extensively examined in gas drug delivery.^[^ ^73^ ^]^ Therapeutic gas‐containing moiety is normally conjugated directly to polymers for responsive gas release.

Direct release of therapeutic gas molecules (such as CO and H_2_S) from polymers is becoming increasingly popular.^[^ ^74^ ^]^ For example, 5‐(4‐Hydroxyphenyl)‐3*H*‐1,2‐dithiole‐3‐thione (ADT–OH) is one of the most widely used H_2_S‐releasing molecules. Poly(ethylene glycol)‐ADT (PEG‐ADT) released H_2_S for anti‐inflammatory effect by firstly reducing ─S─S─ (disulphide bond) and then another quick intrinsic reduction.^[^ ^75^ ^]^ Yang et al. prepared a series of polymer‐NO conjugates that could efficiently release NO.^[^ ^76^ ^]^ In another study, Cheng et al. prepared metal‐free CO‐releasing polymer by direct polymerisation of 3‐hydroxyflavone (3‐HF) derivatives. Herein, 2‐((((4‐(3‐((2‐nitrobenzyl)oxy)‐4‐oxo‐4*H*‐benzo[*g*]chromen‐2‐yl)benzyl)oxy)carbonyl) amino) ethyl methacrylate (FNM) monomer with photosensitive *o*‐nitrobenzyl ether and 3‐HF derivatives was constructed through reversible addition‐fragmentation chain transfer (RAFT) polymerisation. Upon light irradiation, the CO‐releasing amphiphilic compound was assembled internally into micelles in water, and removed *o*‐nitrobenzyl moieties and oxygenated 3‐HF to release CO.^[^ ^77^ ^]^ These types of metal‐free gas‐releasing polymers are gaining interest in recent years due to their biocompatibility, which can help mimic the use of other inorganic materials that may be toxic to biological tissues after gas release.^[^ ^78^ ^]^

Many polymers have been used to formulate gas therapeutic nanosystems for various diseases.^[^ ^79^ ^]^ In future, a focus on polymer carriers that play active roles in releasing gases by their sensitivity to tumour microenvironments (TMEs) or external stimuli to allow effective gas release may assist the use of polymers for therapeutic gas delivery.

### 3.3 Protein/peptide‐modified materials

**FIGURE 6:**
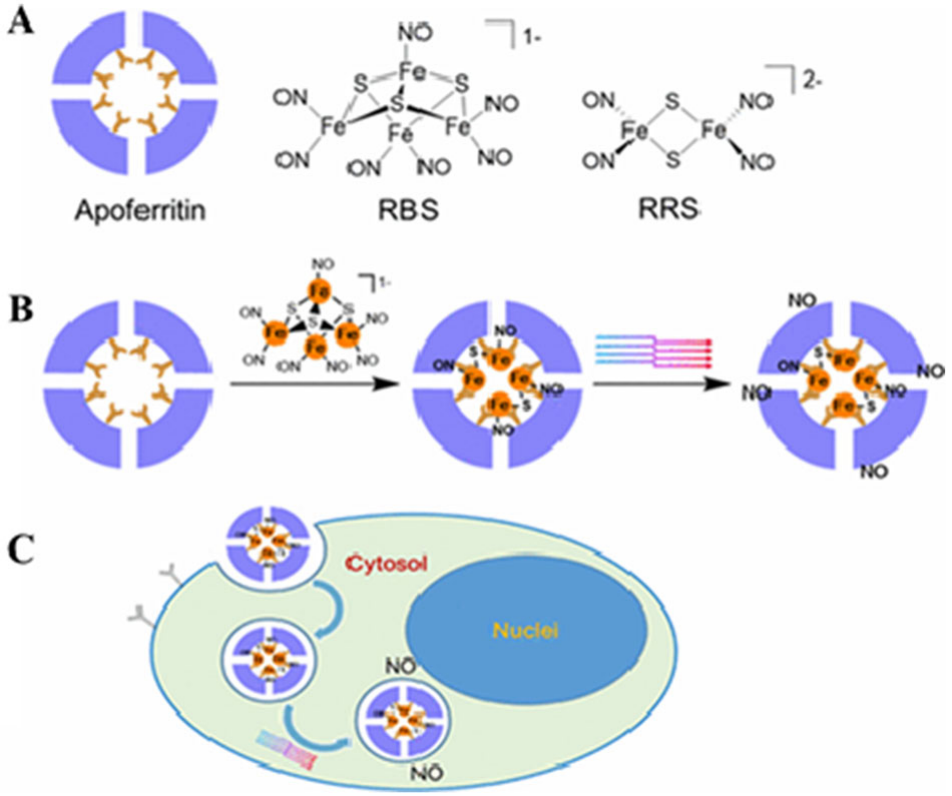
Schematic representation of Apoferritin and chemical structures of RBS and RRS molecules indicating RBS incorporation and NO release. The figure demonstrates intracellular internalisation of nanocomposites and intracellular NO release. Adapted with permission.^[^ ^81^ ^]^ Copyright 2017, American Chemical Society

Peptides have been extensively examined as drug delivery systems for therapeutic and diagnostic purposes.^[^ ^80^ ^]^ These biocompatible peptides/proteins are abundant in nature, and genetically and chemically modifiable during synthesis for therapeutic purposes. Many peptides/proteins have been used as carriers, while others have been exploited to directly release gases. As shown in Figure 6, the cavity of apoferritin (a protein present in the intestinal mucosa membrane) acted as Fe‐depots to maintain iron homeostasis for systemic regulation. Such inner binding sites were used to anchor a variety of metal species such as Fe and Cu complexes, which were used to cage metal‐coordinated complexes as GRMs.^[^ ^81^ ^]^

Some peptide‐mediated gas releasing systems have currently been designed. For example, the chemically labile CORM‐3 was combined with several proteins, such as human serum albumin (HSA), haemoglobin, myoglobin, hen egg‐white lysozyme (HEWL) and human transferrin.^[^ ^82^ ^]^ Studies have demonstrated that CORM‐3 molecules could break down into metallate proteins with Ru(CO)*_x_* fragments, such as [Ru(CO)_2_(H_2_O)_3_]^2+^ and [Ru(CO)(H_2_O)_4_]^2+^, which efficiently released CO.^[^ ^83^ ^]^ Chaves‐Ferreira et al. designed Ru^II^(CO)_2_–protein complexes by combining CORM‐3 with histidine residues of proteins in aqueous solution. They confirmed that the spontaneous release of CO in aqueous solution, cells and the mouse body was instigated by metalloproteins.^[^ ^84^ ^]^ However, most reported protein‐CORM adducts as a large molecule exhibit rapid clearance from the body. Thus alternative strategies for nanoformulations have to be introduced to improve bioavailability at the required delivery sites.

### 3.4 Lipid‐based nanomaterials

Lipid nanoparticles have been used for effective drug delivery for many years. In GT, they help control the release and diffusion of gases in biological tissues.^[^ ^85^ ^]^ For instance, Schoenfisch et al. demonstrated the mediatory role of liposomal drug delivery system containing propyl‐1,3‐propanediamine/NO (PAPA/NO) and diethylenetriamine/NO (DETA/NO) (two *N*‐diazeniumdiolates), which quickly and slowly released NO molecules, respectively. These GRMs released NO spontaneously under physiological conditions and the release was slightly increased at high temperatures and low pH values. Interestingly, NO‐releasing PAPA/NO liposomes had a much slow NO‐release kinetic as compared to unencapsulated or free NO‐releasing molecules.^[^ ^86^ ^]^ This mechanism enhanced the cell apoptosis as a relatively high level of therapeutic gases was continuously exposed to cancer cells after uptake. Similarly, Quin et al. prepared a liposomal H_2_S releasing nanoparticle that incorporated trisulphide linker into the liposome, which mediated thiol‐triggered H_2_S release. Evidently, this trigger ruptured the liposomal membrane to release the generated gas molecules. Most importantly, trisulphide conjugates incorporated into liposomes did not release H_2_S spontaneously, which may be attributed to the fact that H_2_S gases did not fully diffuse across the bilayer lipid membrane as compared to the micelle control.^[^ ^87^ ^]^ It is evident that lipid nanoparticles modulated the released gas amount that is made available for therapeutic purposes at the diseased sites.

### 3.5 Biomimetic materials

Biomimetic materials are derived from naturally occurring sources. They have some specific functions, which can be adapted to deliver various therapeutic agents. Bio‐inspired materials that mimic biological materials include exosomes, high‐density lipoproteins, erythrocytes, virus‐mimicking nanoparticles, bacteria‐like nanoparticles and aptamers. They can be reconstituted or slightly modified in a recombinant process to suit specific drug delivery and release purposes. Some of these materials have been explored in gas release systems to maximise therapeutic effects through synergy. Du et al. hypothesised that an NO‐releasing molecule incorporated in mesenchymal stem cell (MSC)‐derived exosomes boosted the proangiogenic functions of exosomes in cardiovascular conditions. They found that these exosomes increased vascular epithelial growth and modulated angiogenesis.^[^ ^88^ ^]^ In a similar manner, Hou et al. demonstrated that the exosome surface improved their endothelial function, including platelet endothelial cell adhesion molecule‐1 (CD31) expression, cell migration and NO release.^[^ ^89^ ^]^ In another study, Rink et al. synthesised a high‐density lipoprotein (HDL) delivery system that was incorporated with NO molecules. NO was conjugated to DPPTE phospholipid containing thiol group for targeted delivery to cells. This recombinant HDL nanoparticle maintained its cholesterol sequestration property while its combination with NO enhanced the respective vasoprotective effect. HDL nanoparticles targeted scavenger receptor type B‐1 (SR‐B1) expressed by various cells, and therefore enhanced delivery of NO. They subsequently demonstrated the effective release of NO and formation of GSH via concurrent Cu(II) catalysis for atherosclerosis.^[^ ^90^ ^]^

### 3.6 Comparison of nanosystems for GRM delivery and release

Nanocarriers for gas prodrug delivery and gas release have special roles including efficient loading, targeted delivery and controlled release of gases. Delivery vehicles with common characteristics such as size, shape, loading efficiency, targeting strategy and surface chemistry have different levels of efficiency for therapeutic gas delivery.^[^ ^91^ ^]^ While most nanocarriers discussed in this review are capable of loading high amounts of both hydrophilic and hydrophobic GRMs,^[^ ^54^, ^55^, ^60^, ^86^ ^]^ some lipids and polymers are further capable of efficiently modulating gas release and diffusion, which is the most desirable advantage for gas delivery nanomaterials.^[^ ^87^ ^]^ Unlike inorganic nanomaterials, biocompatible polymers, peptides, biomimetic and lipid delivery systems have also shown superior abilities for biofriendly delivery, as some have been approved for commercial use.^[^ ^92^ ^]^ Efficiency in evading the immune system is an additional essential feature, which has also been assessed among these nanoparticles based on previous reports and current gas delivery vehicles.^[^ ^88^, ^93^ ^]^ With these assessments, we try to classify these nanomaterials from highly efficient to least efficient across various capabilities.

As summarised in Table 2, the level of loading, targeting, mediation of gas release, biocompatibility and immune escapability is herein classified and compared based on the evaluations from cited articles in the review. Overall, their specific roles for GT may provide some clues for researchers to consider and choose suitable nanomaterials for effective GT. For example, inorganic nanomaterials are excellent for gas releasing control, but are less biocompatible and less capable of escaping the immune system (Table 2). However, coating inorganic nanoparticles with biomolecules or biopolymers would enhance these properties for efficient therapeutic gas delivery.

**TABLE 2:** Capability assessment of nanomaterial delivery systems for GT

Nanosystem	Proteins/peptides	Inorganic nanomaterials	Polymers	Lipids	Biomimetic
Modulation of release	**	*****	***	****	**
GRM loading	**	****	****	****	***
Targeting	***	***	****	****	****
Biocompatibility	****	*	***	****	*****
Immune escapability	****	*	**	****	****

*Represents the degree associated with the nanosystem, where * is the lowest, and ***** is the highest.

## 4 STIMULI‐RESPONSIVE STRATEGIES FOR GAS RELEASING FROM PRODRUGS

In the early years of discovery, gasotransmitters were administered by direct inhalation, a gold standard used to determine the limit of exposure and the LD_50_ value for various therapeutic gases.^[^ ^94^ ^]^ However, this method is highly unpredictable and its control is extremely challenging. Since most of these gases are suitable for diseases, such as cancer and inflammation, their presence in the blood and other undesirable peripheral tissues may cause severe side effects. So recent strategies have explored intrinsic and extrinsic stimuli to specifically release gases in desirable tissues for therapeutic purposes. Herein, we discuss some novel release strategies that have been recently developed, and some possible future prospects to help steer these methods into clinical use.

### 4.1 Internal stimuli used for gas release

#### 4.1.1 pH–sensitive gas‐releasing

The basic principle for acid‐responsive drug release is very common in cancer drug delivery because of the well‐known acidity nature of TME caused by the preferential conversion of elevated glucose to lactic acid, as propounded by Warburg in 1930. Since diseased cells may require high glucose uptake for energy and metabolism, this conversion is commonly produced inside tumour cells. The acidity in TME and the hyperacidity in gastrointestinal tract have been harnessed to release different kinds of drugs^[^ ^95^ ^]^ via bond‐breaking or dissociation. For instance, hydrogen atom in thiols (R‐SH) can be replaced by NO as S‐NO molecules in highly acidic media (pH < 4).^[^ ^96^ ^]^ The reduction of S‐NO compounds in acidic medium (e.g. low pH in TME) leads to gradual NO release. Various non‐metallic CORMs and H_2_S‐releasing molecules have been designed to trigger gas release in acidic conditions. Kang et al. prepared H_2_S‐releasing molecules from phenylphosphonothioic dichloride that released H_2_S via an intramolecular cyclisation upon exposure to weakly acidic conditions (pH 5 and 6).^[^ ^97^ ^]^

**FIGURE 7:**
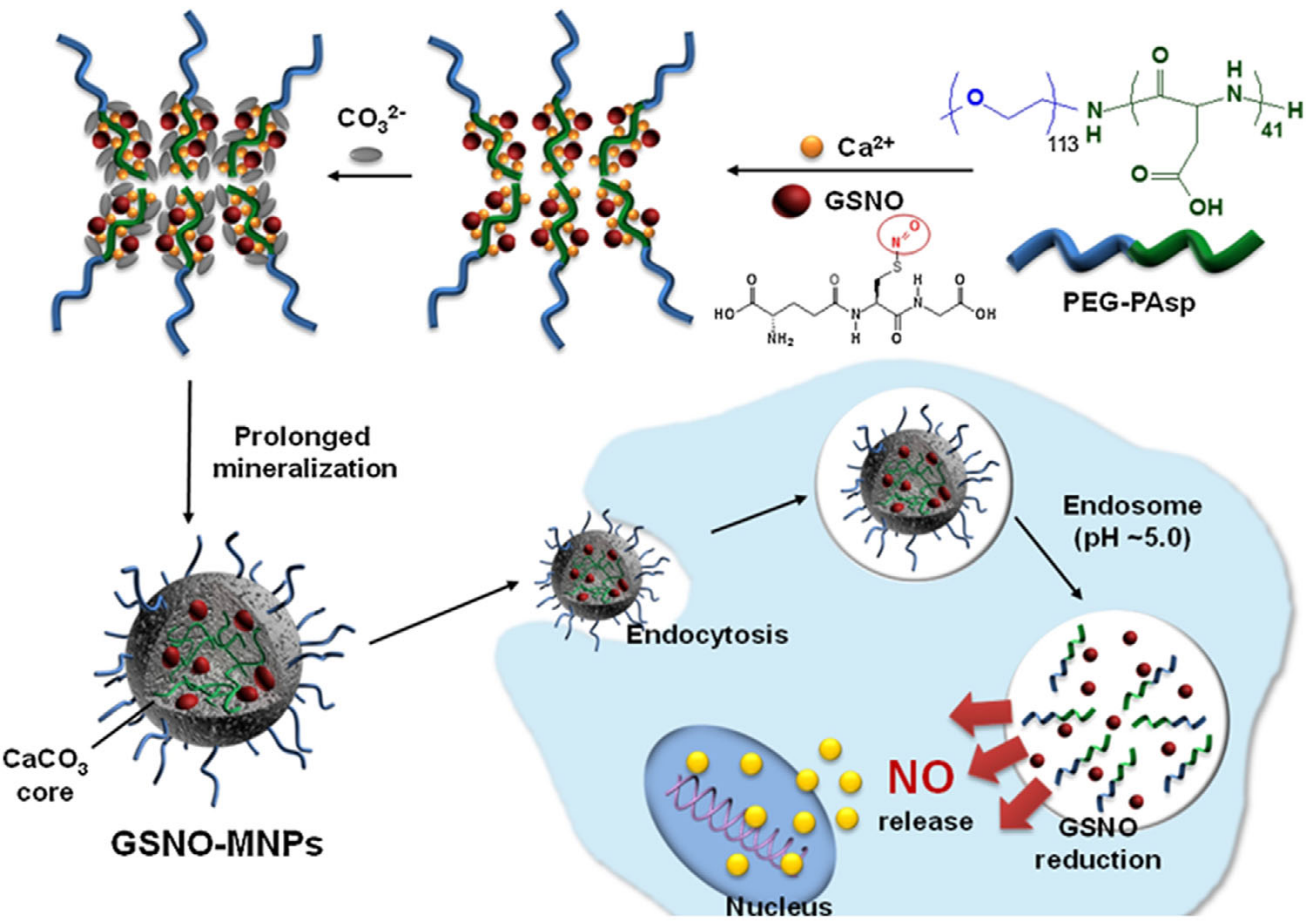
Schematic illustration of block copolymer‐template of GSNO‐loaded CaCO_3_‐mineralised nanomaterials and intracellular NO release. Adapted with permission.^[^ ^98^ ^]^ Copyright 2016, Elsevier

In a similar way, some pH‐sensitive nanomaterials have been employed to successfully shuttle drugs to target sites, where they can be dissolved by acids to expose the drugs. Lee et al. designed a pH‐sensitive system capable of collapsing under acidic conditions to stimulate the release of S‐nitrosoglutathione (GSNO).^[^ ^98^ ^]^ In their work, they used a non‐toxic biomineral, calcium carbonate (CaCO_3_) that maintained the crystalline structure at neutral pH, but dissolved into ionic species in acidic environments such as in TME (pH 7.0∼6.0), endosomes (pH 6.0∼5.0) and lysosomes (pH 5.0∼4.0). As shown in Figure 7, this process collapsed the drug delivery system in situ and released GSNO, and the acid subsequently released NO from GSNO only in TME and cancerous cells slowly.^[^ ^98^ ^]^ Chung et al. also developed an injectable PLGA hollow microsphere (HM) nanosystem loaded with irinotecan (CPT‐11) and NONOate, an NO‐releasing molecule. In acidic tumour tissues, environmental protons infiltrate the shell of HMs and react with encapsulated NONOate molecules to release NO and reverse the Pgp‐mediated multidrug resistance (MDR).^[^ ^99^ ^]^ Gas delivery systems that rely on acidity is one of the most reliable strategies since all kinds of cancers are associated with low pH as compared to healthy cells. This may help reduce the non‐specificity issues.

#### 4.1.2 Enzyme‐triggered gas releasing

Enzymes are biological molecules capable of recognising substrates and performing specific catalytic reactions. These intrinsic enzymes are usually esterases or proteases with specific recognition for various molecules.^[^ ^100^ ^]^ Drug delivery scientists have made efforts to take advantage of the action of these enzymes that are conspicuously generated at various disease sites.^[^ ^101^ ^]^ The gas‐releasing prodrugs can be catalytically lysed by synthesised biomimetic enzymes in the diseased environments to release the gas molecule as well. Romanski et al. produced acyloxybutadiene tricarbonyl iron complexes as enzyme‐triggered CORMs (ET‐CORMS). In gas releasing molecules, the structural reliance on the position of the esters in cyclohexanone or cyclohexanedione is utilised to bestow a specific functionality that esterases present in biological cells can recognise and catalyse to trigger CO release.^[^ ^102^ ^]^ The use of cytochromes, esterase, oxireductase, nitroreductase, glutathione peroxidase, β‐galactosidase (β‐gal) and glycosidase for NO release is also well documented.^[^ ^103^ ^]^ Further, H_2_S release is also demonstrated in a series of isomeric caged‐carbonyl sulphide (COS) compounds such as thiocarbamates, thiocarbonates and dithiocarbonates, which are enzymatically catalysed to release COS for quick conversion to H_2_S by the ubiquitous enzyme carbonic anhydrase.^[^ ^104^ ^]^

**FIGURE 8:**
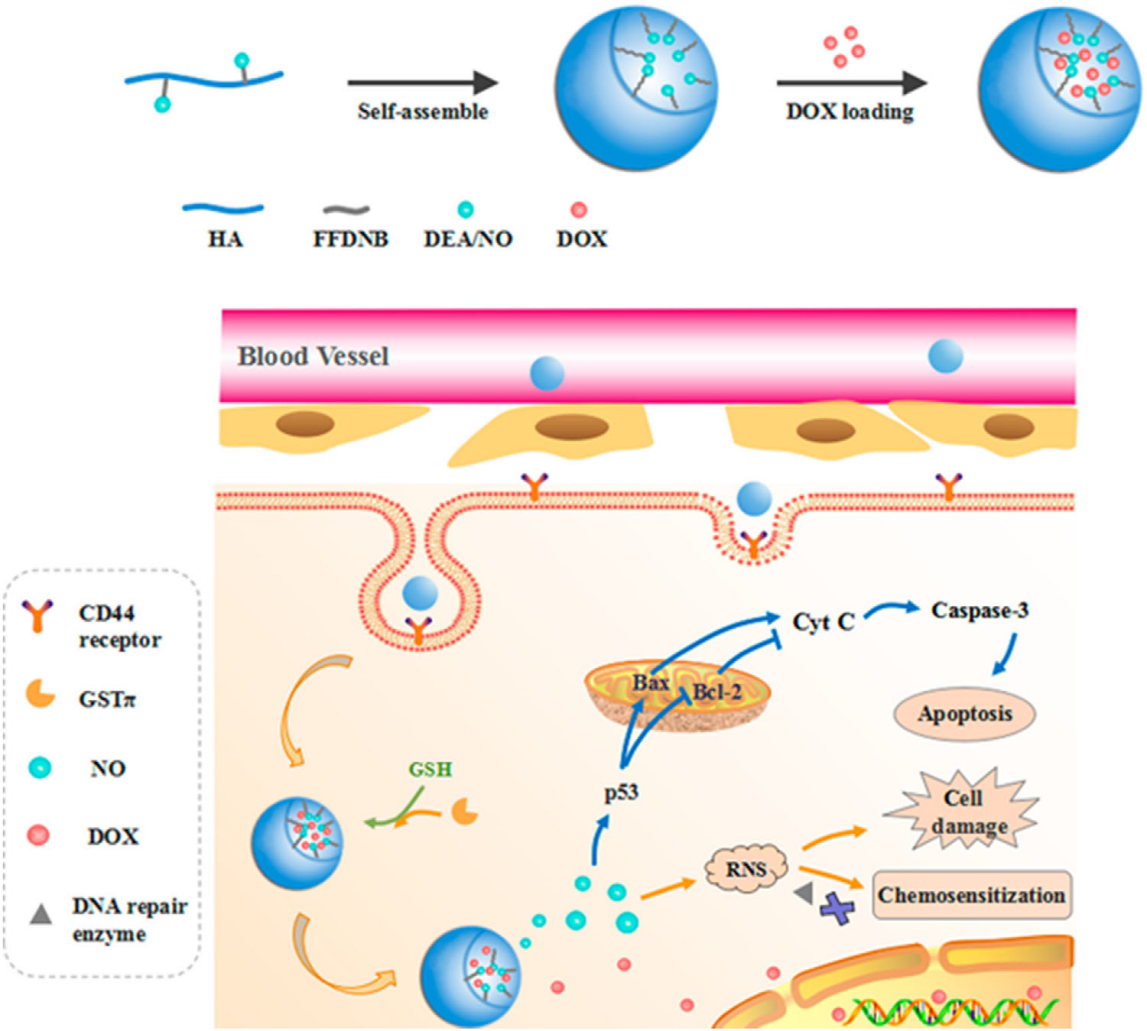
Scheme of enzyme‐triggered NO release, enhanced tumour targeting and cancer therapy. Adapted with permission.^[^ ^105^ ^]^ Copyright 2021, American Chemical Society

Similarly, various prodrugs can be loaded into nanocarriers to achieve this purpose with enhanced efficiency. For example, Hou et al. produced an intracellular enzyme‐sensitive NO release nanosystem to achieve cancer cell cytoplasm‐specific disruption of encapsulated diethylamine NONOate (DEA/NO) and doxorubicin (DOX).^[^ ^105^ ^]^ In this work, they conjugated the NO‐releasing molecule to hyaluronic acid (HA) to self‐assemble as micelles (DOX@HA‐DNB‐DEA/NO) (Figure 8). HA receptor is supposed to mediate internalisation into cancerous cells, wherein intrinsic hyaluronidase catalyses HA into small conjugate pieces. On the other hand, DEA/NO responds to the overexpressed glutathione S‐transferase π, leading to NO release.^[^ ^105^ ^]^ The current challenge for this technique is to identify the specific types of enzymes present in various kinds of diseased tissues and cells that are absent in healthy cells. Prospective studies can be done to quantify and detect specific enzymes for different kinds of diseases to maximise the use of this technique for therapeutic gas release.

#### 4.1.3 Reduction‐sensitive gas release systems

Most diseased tissues have specific characteristics that could be exploited to release gas molecules by cleaving the specific bond in gas releasing molecules.^[^ ^106^ ^]^ For example, redox reactions involve the cleavage of various bonds by intrinsic redox agents to release gas in a responsive manner. The reducing environment in tumour tissues is usually dictated by the reduction and oxidation states of NADPH/NADP^+^ and glutathione (GSH, GSH/GSSG), which are produced and transported from the cytosol to the mitochondria in the presence of oxidative stress in diseased cells.^[^ ^85a^ ^]^ This intrinsic redox phenomenon is produced when the concentration of GSH is higher than NADPH in TME (about 4‐fold higher than in normal tissues). The elevated GSH level plays a critical role in breaking diverse disulphide bonds,^[^ ^107^ ^]^ and there have been several nano‐based systems, which are highly responsive to GSH for gas release.^[^ ^108^ ^]^ Redox‐responsive groups including thiol (─SH), disulphide bond (─S─S─), diselenide bond (─Se─Se─), platinum conjugation (─Pt─), thioether bond (─S─) are often used in this technique.^[^ ^109^ ^]^ For example, diiron hexacarbonyl complex [Fe_2_(μ‐SCH_2_CH(OH)CH_2_(OH))_2_(CO)_6_] (TG‐FeCORM) produced by Gao et al. released CO by GSH in the presence of cysteamine (CysA).^[^ ^110^ ^]^ Diallyl trisulphides (DATS) released H_2_S upon exposure to GSH (2 mM).^[^ ^111^ ^]^ An MSN‐based GSH‐triggered system was also fabricated to load high amounts of DATS,^[^ ^112^ ^]^ with H_2_S release peaking at 4 h in PBS.

**FIGURE 9:**
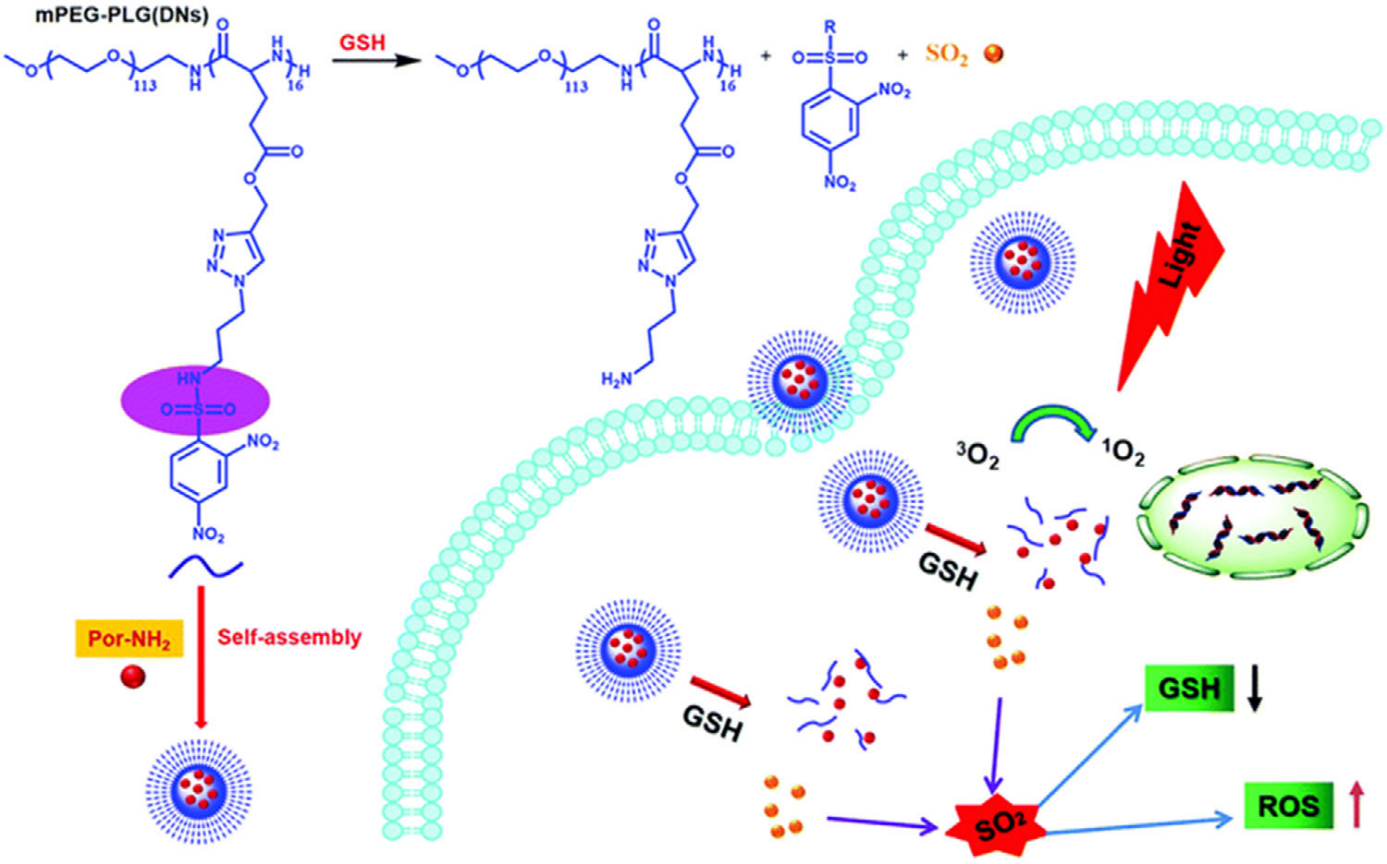
Schematic illustration indicating the preparation of NP‐DN‐Por, GSH‐triggered SO_2_ release and enhanced photodynamic therapy. Adapted with permission.^[^ ^19a^ ^]^ Copyright 2018, Elsevier

Interestingly, Zhang et al. prepared an amphiphilic polymer prodrug (mPEG‐PLG(DNs)) through conjugation of SO_2_‐release prodrug, *N*‐(3‐azidopropyl)‐2,4‐dinitrobenzenesulphonamide (AP‐DNs) to the methoxy poly(ethylene glycol)‐*block*‐poly(γ‐propargyl‐l‐glutamate) (mPEG‐PPLG) polymer backbone.^[^ ^113^ ^]^ The synthesised mPEG‐PLG (DNs) encapsulated 5‐(4‐aminophenyl)‐10, 15, 20‐triphenylporphyrin (Por‐NH_2_) to form GSH‐responsive nanoparticles capable of releasing SO_2_ via the overexpressed GSH in tumour cells. As seen in Figure 9, dinitrobenzenesulphonamide group released SO_2_ in response to abundant GSH in cancer cells.^[^ ^19a^ ^]^

#### 4.1.4 Oxidation–induced gas release systems

The heterogeneity of various diseases including cancer, arthritis and diabetes gives rise to the overproduction of reactive oxygen species (ROS), a by‐product of oxidative phosphorylation (a source of energy production in cells), which can be utilised to release gas molecules for therapeutic purposes.^[^ ^114^ ^]^ Hydrogen peroxide (H_2_O_2_) is often produced by high levels of superoxide dismutase in diseased cells through transformation of superoxide ions generated in mitochondria, and thus H_2_O_2_ is often used to orchestrate the release of various payloads for cancer treatment.^[^ ^115^ ^]^ For instance, Ayudhya et al. prepared amine‐carboxyboranes prodrugs for CO release upon exposure to H_2_O_2_^[^ ^116^ ^]^ and Chauhan et al. produced carbamothioates to tuneably release H_2_S after activation by ROS.^[^ ^117^ ^]^

**FIGURE 10:**
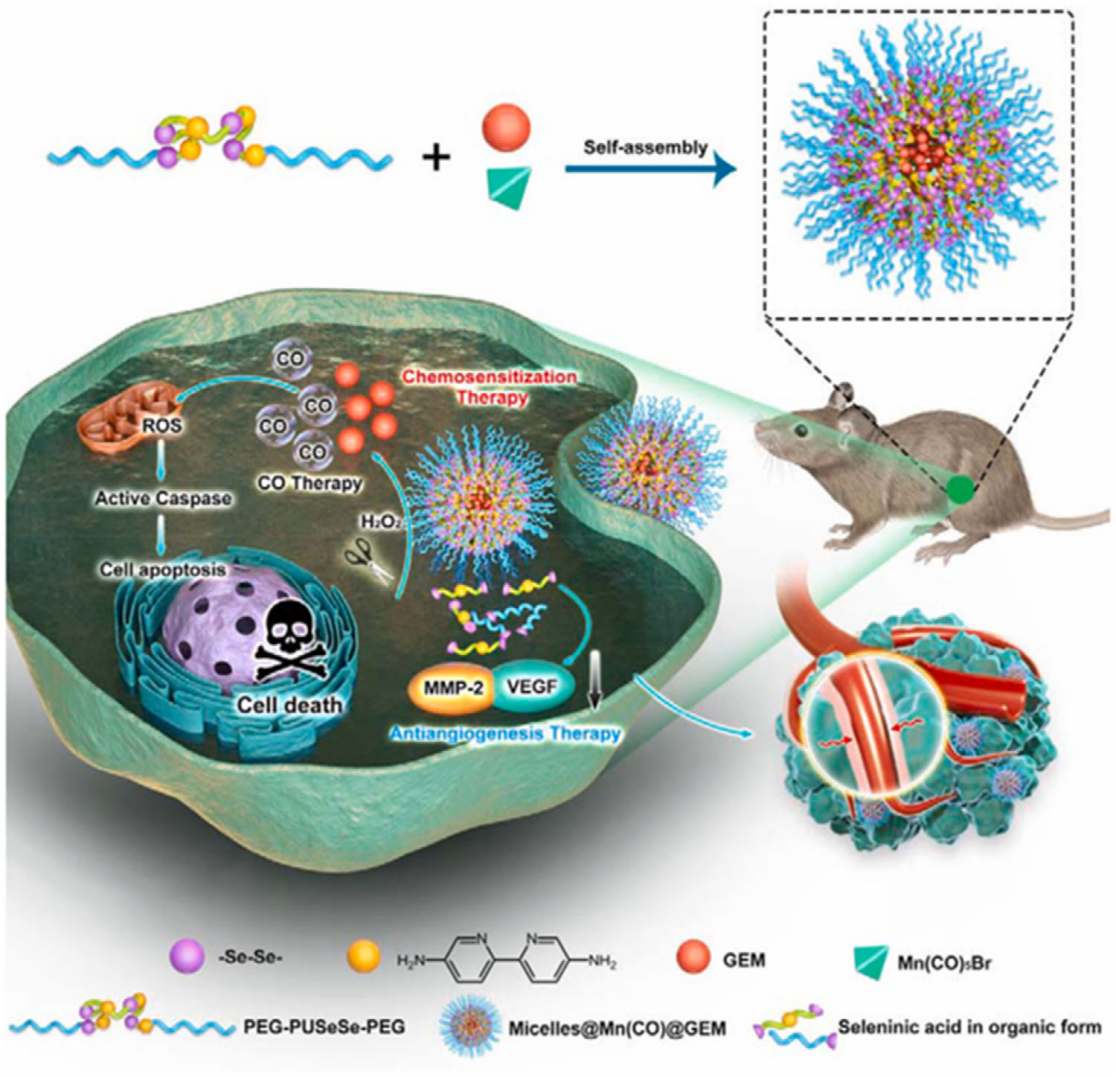
H_2_O_2_‐responsive CO release from Mn(CO)‐containing micelles via Fenton‐like reaction in situ. Adapted with permission.^[^ ^120^ ^]^ Copyright 2021, Elsevier

In a more recent study, Jin et al. loaded manganese carbonyl (Mn_2_(CO)_10_, MnCO) in hollow mesoporous silica nanoparticle (hMSN) to passively target cancer cells.^[^ ^118^ ^]^ The study suggests that MnCO reacts with over‐generated H_2_O_2_ in the tumour milieu to liberate CO gas internally via a new Fenton‐like reaction. H_2_O_2_ supposedly enters the mesoporous pores of MnCO@hMSN and is converted to strongly oxidative •OH radicals under the catalytic action of Mn^2+^. The •OH radicals subsequently oxidise Mn in MnCO and OH^–^ ions competitively coordinate with the Mn centre, causing CO release. In a similar study, an erythrocyte membrane gas delivery system (MGP@RBC) was prepared to amplify the internal generation of CO for combined cancer starvation and GT.^[^ ^119^ ^]^ The CO gas was released by over‐generated H_2_O_2_ in TME. This nanosystem was designed by encapsulating glucose oxidase (GOx) and Mn_2_(CO)_10_ (CORM) into the biocompatible polymer poly(lactic‐*co*‐glycolic acid), which was further covered by red blood cell (RBC) membrane. The concomitant generation of H_2_O_2_ efficiently triggered CO release to cause dysfunction of mitochondria and activate caspase, thereby resulting in apoptosis of cancer cells. Similarly, Yang et al. prepared H_2_O_2_‐responsive Mn(CO)_5_Br‐containing micelles for CO release (Figure 10). They also utilised a Fenton‐like reaction to rapidly release CO in situ. The CO/chemosensitisation/antiangiogenesis synergistic therapy strategy has exhibited favourable anti‐tumour efficacy with good biocompatibility.^[^ ^120^ ^]^

### 4.2 External stimuli‐responsive gas‐releasing

#### 4.2.1 Photo‐responsive gas release systems

There are several approaches for photon‐responsive gas releasing employing various light sources, including UV, visible and near infrared (NIR) light. Generally, photon‐induced GT is performed across different wavelengths from UV–vis to NIR. As most therapeutic gas molecules are strongly bonded and can be more easily released upon triggering with UV or visible light, thus a few UV–vis light responsive GRMs have been developed for gas release. Hu group developed various NO responsive systems using *N*‐nitrosoamine‐based NO donors that were polymerised into amphiphiles using reversible addition‐fragmentation chain transfer (RAFT).^[^ ^121^ ^]^ The NO‐releasing amphiphiles self‐assembled into micelles and selective NO release was achieved by irradiating micelle solution with visible light (410 nm) for bacteria killing. It is well known that UV–vis light has limited tissue penetration and may cause some damages to tissues. Therefore, delivery nanosystems carrying gas release molecules are further designed to employ NIR as the trigger as NIR light can penetrate much deeper tissues. Fortunately, there are agents that can transfer NIR to UV–vis photons to trigger gas release. For instance, Opoku‐Damoah et al. recently prepared a versatile nanosystem capable of shuttling photo‐responsive CORMs for targeted cancer therapy.^[^ ^79^ ^]^ The core‐shell upconversion nanoparticles (UCNPs) converted biocompatible low‐energy NIR (808 or 980 nm) to ultraviolet (UV) light (360 nm) to trigger CO release from the CORM. The synthesised nanoplatform instigated quick release of CO upon 808 or 980 nm NIR light excitation. The optimised folate‐endowed nanosystem was more efficiently internalised by HCT116 cancer cells with dose‐dependent cytotoxicity. In a similar study, SO_2_ prodrug‐loaded rattle‐structured upconversion@silica nanoparticle (RUCSN) was constructed to convert NIR to UV light for SO_2_ generation.^[^ ^122^ ^]^ SO_2_ prodrug‐loaded RUCSNs showed high cell uptake, good biocompatibility, and high NIR light‐triggered cytotoxicity. Furthermore, cytotoxic SO_2_ induced cell apoptosis by increasing the intracellular ROS level and damaging nuclear DNA.

**FIGURE 11:**
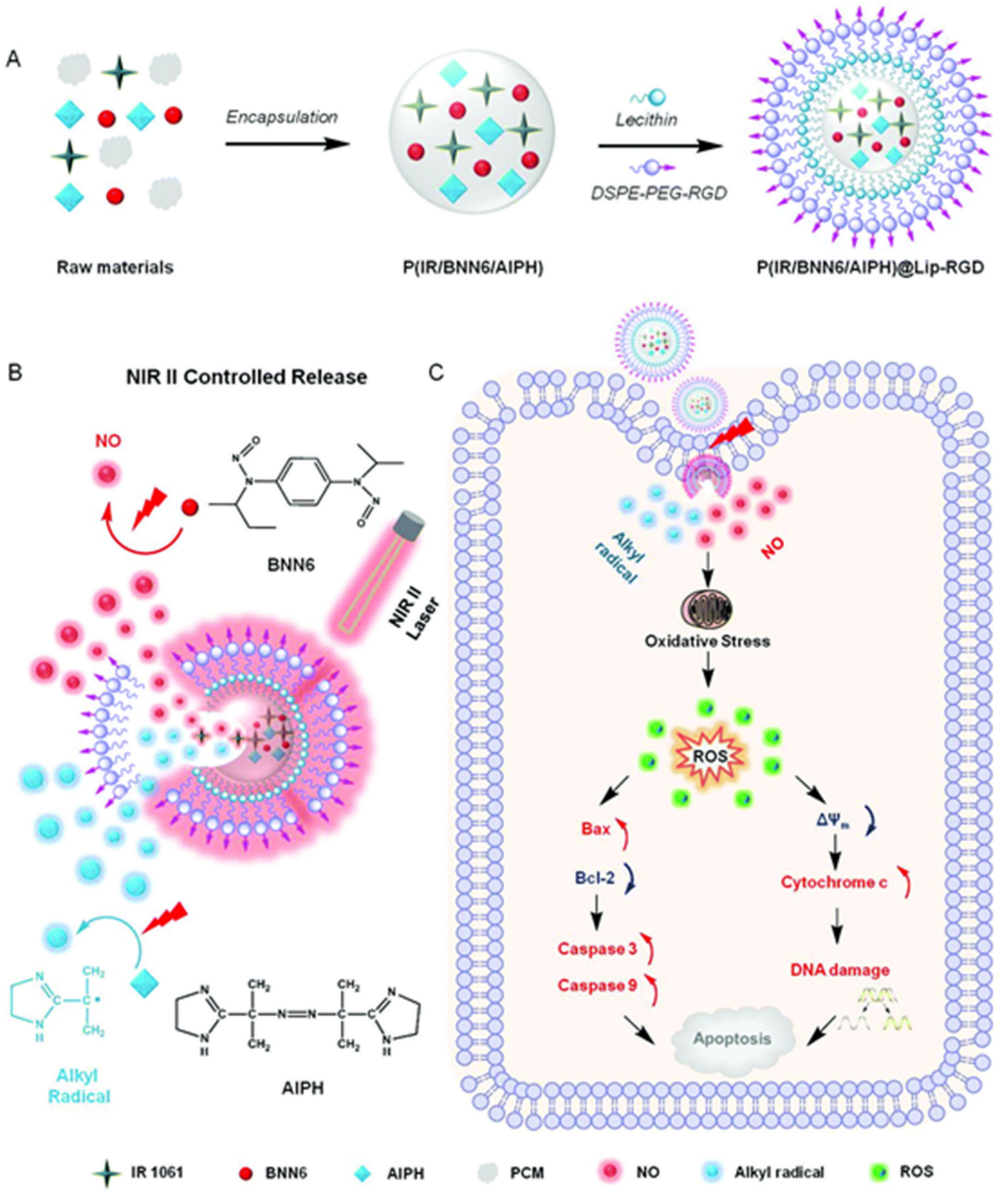
Synthesis of P(IR/BNN6/AIPH)@Lip‐RGD using a two‐step method with synergistic alkyl radical and NO anticancer mechanism under NIR II laser irradiation. Adapted with permission.^[^ ^125^ ^]^ Copyright 2021, RSC Pub

On the other hand, photothermal conversion transfers photon energy to thermal energy to stimulate gas release.^[^ ^123^ ^]^ A lot of photon‐responsive materials are derived from plasmonic metal compounds with a relatively high response in the NIR region.^[^ ^3a^ ^]^. Dong et al. developed photothermal cancer GT using diketopyrrolopyrrole (DPP) derivatives as an efficient photon‐heat converter.^[^ ^124^ ^]^ NO‐release molecule (4‐nitro‐3‐trifluoromethylaniline, NF) and dimethylaminophenyl were covalently bonded to the light‐responsive DPP core (denoted as DPP‐NF). This nanoplatform achieved controllable “on–off” release of NO under light or dark conditions. DPP‐NF NPs increased the temperature to above 50°C under irradiation (660 nm, 0.8 W cm^–2^), making themselves superior to other reported DPP derivatives. This work has demonstrated that the controllable photothermal NO release could trigger cancer cell death and also overcome photodynamic therapy (PDT) inefficiency limited by hypoxia in TMEs.^[^ ^124^ ^]^ In another recent study, Wu et al. designed an NIR II laser‐induced NO release system containing BNN6 and alkyl radical (an initiator) with lipids and peptide nanomaterials for tumour homing and mitochondria‐mediated cancer GT.^[^ ^125^ ^]^ The photothermal effect led to NO release and changed the lipid phase of the nanosystem and cell membrane (Figure 11).

#### 4.2.2 Ultrasound‐responsive gas delivery systems

Ultrasound (US) has been widely used for stimulus‐responsive drug delivery for decades,^[^ ^126^ ^]^ such as triggering both mechanical and thermal phenomena via cavitation, streaming and hyperthermia.^[^ ^127^ ^]^ Ultrasound transducers are used to produce longitudinal pressure waves that can be transmitted into the body at varying frequencies (0.1–50 MHz).^[^ ^128^ ^]^ In comparison with light‐mediated release approach, US has a much deep penetration through a variety of tissues and can interrupt the cell membrane in a non‐invasive pattern without dramatic energy dissipation.^[^ ^129^ ^]^

Based on these advantages, several ultrasound‐responsive NO release systems have been constructed. For example, Chen et al. prepared hollow mesoporous silica nanoparticles (HMSNs) with the surface modified with poly(ethylene glycol) (PEG) polymer^[^ ^130^ ^]^ and loaded L‐arginine (LA) as a natural NO prodrug in HMSN mesopores. The focused ultrasound activated H_2_O_2_ to generate ROS and oxidised LA via the energy transformation, releasing NO.^[^ ^130^ ^]^ Alghazwat et al. developed an ultrasound‐responsive micelle for responsive CO delivery.^[^ ^131^ ^]^ The micelle was constructed with the pluronic shell encapsulating CO‐releasing molecule (CORM‐2) as the core. The localised release mechanism is based on the pluronic response to ultrasound, and the subsequent reactions between CORM‐2 and cysteine, releasing CO without significantly breaking the micelle. The nanosystem was used to treat prostate cancer cells (PC‐3), and cell apoptosis was observed 24 h post‐treatment.^[^ ^131^ ^]^ Although many of these nanomedicines are in early stages of development, the non‐invasive nature of US makes it a promising option for future clinical use.

#### 4.2.3 Magnetics‐responsive gas release systems

Magnetic nanoparticles (MNPs) have been used extensively in drug delivery and diagnostics,^[^ ^132^ ^]^ and superparamagnetic iron oxide nanoparticle (SPION) is one of the most practical stimuli‐responsive drug delivery systems.^[^ ^3a^ ^]^ Magnetic hyperthermia is often used to trigger gas release and magnetic nanoparticles can be directed to the diseased sites with an external AC magnetic field.^[^ ^133^ ^]^

Meyer et al. reported a magnetic iron oxide nanoparticle (IONP)‐based system, oximeCORM@IONP, by assembling oxime‐based CO‐releasing molecules (oximeCORMs) with the catechol‐modified backbone of IONPs for CORM delivery.^[^ ^134^ ^]^ Through local magnetic heating with an external alternating magnetic field, CO release was instigated.^[^ ^134^ ^]^ Most of such release systems are related to heat generation by magnetic action to trigger the release of gaseous molecules. However, this type of system has not been extensively explored in gas delivery because of the inefficiency of thermal‐induced bond cleavage.

### 4.3 Multi‐responsive gas release systems

**FIGURE 12:**
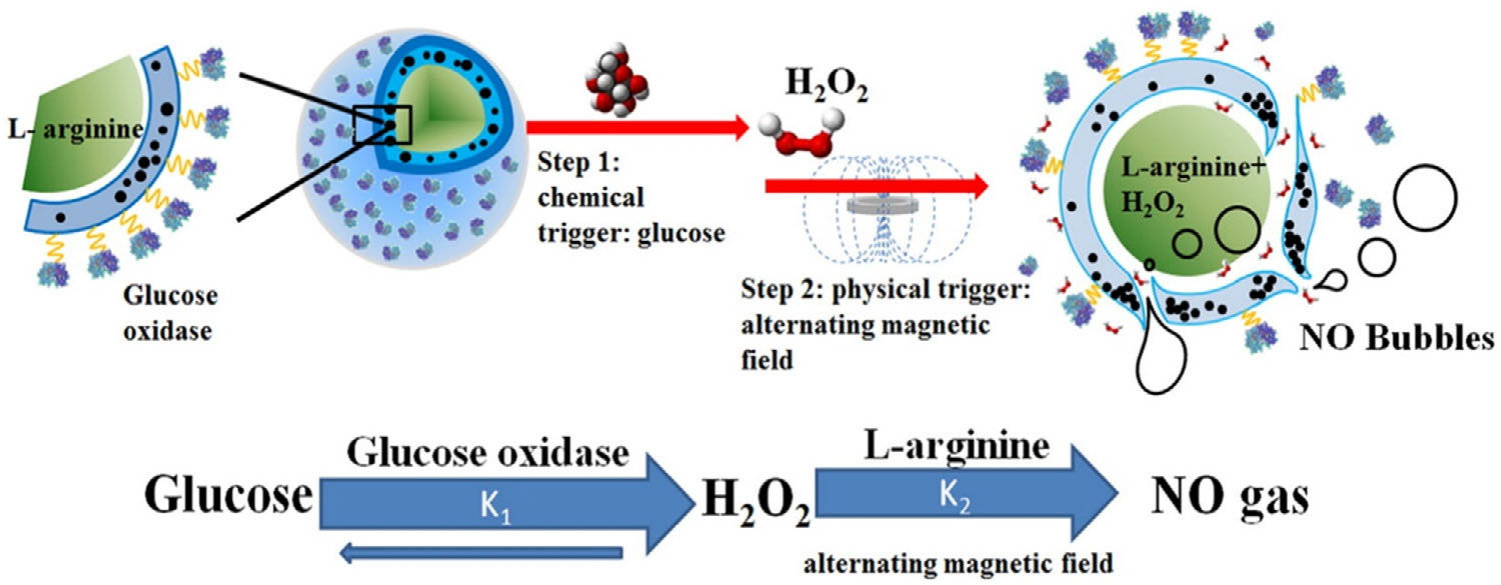
Schematic diagram of microvesicle‐encapsulated magnetic nanoparticles and GOx for dual‐stimuli responsive programmable delivery model. Reproduced with permission.^[^ ^135a^ ^]^ Copyright 2016, Elsevier

The complexity of cancer and its longstanding treatment resistance make it complicated and inefficient to treat cancer by a single therapeutic strategy, and multi‐modal therapeutic nanomedicines are developed for enhanced cancer therapy. Similarly, researchers have smartly combined both intrinsic and extrinsic stimuli to compensate for each other for synergistic gas cancer therapy. In fact, dual‐ and tri‐modal responsive systems have been developed with higher efficiency for gas release.^[^ ^135^ ^]^ Notable examples are all actively based on the stimuli discussed previously. For instance, Yang et al. developed a microvesicle delivery platform that actively responded to three stimuli, that is, GOx, H_2_O_2_ and the external magnetic field, to release NO from L‐arginine (NO pro‐drug).^[^ ^135a^ ^]^ As shown in Figure 12, magnetic nanoparticles were located on the shell, L‐arginine in the inner core, and GOx assembled on the surface. GOx is supposed to catalyse glucose oxidation with O_2_ into gluconic acid and H_2_O_2_. The application of alternating magnetic field generates heat and increases the porosity of the polymer shell, resulting in reactions between L‐arginine and H_2_O_2_ to release NO much more efficiently.^[^ ^135a^ ^]^ Hu et al. prepared a multi‐responsive gas release platform with enzyme inducing nanoparticle shrink and laser inducing NO release.^[^ ^135b^ ^]^ They employed ICG as a photothermal‐responsive sensitiser and conjugated hyaluronic acid (HA) with 2‐(Nitrooxy) acetic acid to form HA‐modified NO prodrug (HN). The upregulated amount of hyalurodinase in cancer tissues shrunk the nanoparticle and NIR light induced hyperthermia for NO release.^[^ ^135b^ ^]^ In another study, An et al. developed a biocompatible nanoplatform with dual pH and ultrasound responses for combined NO GT.^[^ ^136^ ^]^ This nanoplatform was designed with a pH responsive zeolite imidazole framework‐8 (ZIF‐8) containing nitrosoglutathione (GSNO) and chlorin e6 (Ce6) and coating with homologous tumour cell membranes for cancer targeting. In this nanosystem, ZIF‐8 supposed to degrade under acidic conditions to expose GSNO, which is triggered to release NO by ultrasound irradiation simultaneously.^[^ ^136^ ^]^

It has been proven that these novel multiple‐responsive systems are better positioned in preparing efficient delivery systems for gas treatment of various diseases. Such systems ensure that the delivery of prodrugs and release of therapeutic gas molecules are achieved in a concerted manner. More systems that utilise different responses concurrently are encouraged for future clinical studies.

### 4.4 Comparison of gas‐releasing stimuli

Various stimuli‐responsive systems are developed and applied for therapeutic gas release, and each triggering approach has its own advantages and limits. While gases are required in the desired concentration window and at the diseased sites, inefficient release and slow release rate may affect the final therapeutic effect. In this review, we have documented various stimuli‐responsive systems for gas delivery and used this information to evaluate the level of efficiency in terms of gas release, specificity, biocompatibility, tissue penetration and targetability. These evaluations consider the possibility of gas release and physicochemical characteristics of stimuli with the comparable quantity in similar diseases and systems. The release efficiency, tissue penetration and specificity of these stimuli is generally very high among controllable external stimuli (except for UV–vis). However, among the intrinsic stimuli, pH appears to release gases more efficiently than redox and enzymes.^[^ ^19^ ^a,^ ^105^, ^119^, ^120^ ^]^ As compared to extrinsic stimuli systems, these intrinsic stimuli are highly biocompatible, while their driving force within tissues appears not to be strong enough for gas release (<50% compared to magnetic, light and ultrasound stimuli), which usually requires further external stimuli to fully release gases.^[^ ^98^, ^135^ ^]^ External stimuli are generally target‐specific and the power can be adjusted to increase their efficiency, penetration and targetability. Based on the previous literature and our understanding, we have thus tried to provide the possible levels of efficiency (* for least effective and ***** for most effective) among these stimuli discussed.

Table 3 qualitatively compares the various types of stimuli and their performance in terms of various characters relevant to the GT. These evaluations are based on our understanding, and may be not so accurate, so they may just provide some clues for readers to refer to in their GT research. Overall, within the nanosystems reviewed here, magnetic, ultrasound and NIR triggers are preferred because of their superior characteristics (Table 3). Some critical challenges for intrinsic stimuli include a low driving force within tissues, leading to limited release of gases. Therefore the response of nanosystems to pH, redox and enzymatic action needs improvement to be more sensitive and ensure effective release of gases. Further improvement can be achieved by combining site‐specific external stimuli such as ultrasound, light or magnetic field. However, some of these external stimuli have also been known to cause moderate to high irritation within biological tissues at high powers. Aside from these limitations, further experimentation is required to help increase the chances of using these stimuli for clinical GT.

**TABLE 3:** Comparison of gas‐releasing stimuli in important aspects for GT

Stimuli	pH	Redox	Enzyme	Magnetic	UV–vis	NIR	Ultrasound
Release efficiency	***	**	**	****	****	****	*****
Specificity	***	***	*****	**	**	***	***
Biocompatibility	*****	****	****	****	**	***	***
Deep tissue penetration	***	***	***	*****	*	****	*****
Targetability	**	**	**	*****	***	****	****

*Represents the degree associated with the stimulus, where * is the lowest, and ***** is the highest.

Table 4 further summarises various release nanoplatforms and techniques for gas delivery and release stimuli developed in these years. These nanoparticles indicate the therapeutic strategy and the possible nanomaterials that have gained prominence for GT because of their specific gas‐releasing mediatory roles. In general, there are many investigations for NO therapy while the research on H_2_S and SO_2_ nano‐based therapy is relatively limited.

**TABLE 4:** Summary of recent novel therapeutic gas‐releasing nanosystems

Gas	Release strategy	Nanomaterials	Ref.
NO	pH	Liposomes	[137]
		Poly(amidoamine) (PAMAM) dendrimers	[138]
		N‐(2‐hydroxypropyl)methacrylamide (HPMA) polymer	[139]
		CaCO_3_, PEG‐Poly(l‐aspartic acid)	[98]
		PLGA hollow microsphere (HM)	[99]
	GSH	Chitosan	[140]
		Amphiphilic floxuridine (FdU)‐poly ε‐caprolactone (PCL) polymer	[108]
		Poly(2‐diisopropylamino)ethyl methacrylate (pDPA‐MA), galactose	[141]
		Poly(2‐aminoethyl methacrylate hydrochloride‐*co*‐*N*,*N*‐bis(acryoyl)cysttamine‐*co*‐ethyleneglycol dimethacrylate‐*co*‐4‐vinylphenylbronic acid), poly(ethyleneglycol dimethacrylate‐*co*‐2‐(ethylamino) ethyl methacrylate)	[142]
	Enzyme	Hyaluronic acid (HA)	[105]
	ROS	Hollow mesoporous organosilica nanoparticle (HMON)	[143]
	Metal ions	Hyperbranched polyesters	[76b]
		Poly(lactic‐*co*‐glycolic acid) (PLGA)	[144]
		Polyurethane (SP‐60D‐60)	[66]
		Silica nanoparticles	[67]
	Light	Methyl‐β‐cyclodextrin (M‐β‐CD), upconversion nanoparticles	[61]
		*N*,*N*′‐Bis(acryloyl)cystamine (BAC), *N*‐isopropyl acrylamide polymer nanospheres	[145]
		Nitrogen‐bonded graphene oxide	[146]
		Lipids‐RGD, NIR 1061	[125]
		Dendritic Fe_3_O_4_@Poly(dopamine)@PAMAM	[147]
		Tween‐20, upconversion nanoparticles	[147b]
		Polydopamine, galactose‐modified d‐α‐tocopheryl polyethylene glycol 1000 succinate (TPGS)	[57]
		Tween‐Bi_2_S_3_	[148]
		Aza‐BODIPY framework, pegylated lipids	[23a]
		Magnesium silica (MgSiO_3_), lanthanide upconversion nanoparticles	[149]
		Ag_2_S@Bovine serum albumin (BSA)	[60]
		SiO_2_, upconversion nanoparticles Apoferritin	[81]
	Ultrasound	Zeolite imidazole framework‐8	[136]
		Mesoporous silica nanoparticle (hMSN), poly (ethylene glycol) (PEG)	[130]
		PEG‐b‐PCL diblock copolymer	[150]
	Magnetic	Polyvinyl alcohol (PVA) polymer Fe_3_O_4_ superparamagnetic oxide	[135a]
	No stimuli	CarboSil 20 80A polymer	[151]
		Fmoc‐dipeptide hydrogels	[152]
		Poly(n‐butyl methacrylate)	[153]
		Chitosan	[154]
		Capric acid (CA) and octadecane	[155]
		Carboxyl‐functionalised mPEG‐PLGH‐thiobenzamide	[156]
		Mesenchymal stem cell (MSC)‐derived exosomes	[88]
		High‐density lipoproteins, gold nanoparticles	[90]
		Lipids	[86]
CO	ROS	Mesoporous silica nanoparticle (hMSN)	[118]
		Diselenide‐containing diamino‐2, 2′‐bipyridine polymer micelles	[120]
		Erythrocyte membrane	[119]
	Light	Lipid/cholesterol mixtures, lanthanide upconversion nanoparticles	[62]
		Dimethylaminophenyl and diketopyrrolopyrrole	[134]
		Tin disulphide (SnS_2_) nanosheets, polyethylene glycol (PEG)	[70]
		Prussian blue, poly (allylamine hydrochloride), polyacrylic acid, polyethylene glycol amine.	[54]
		Ag_3_PO_4_ doped carbon‐dot‐decorated C_3_N_4_ nanoparticles, histidine rich peptides	[69]
		Mesoporous polydopamine	[157]
		2‐((((4‐(3‐((2‐nitrobenzyl)oxy)‐4‐oxo‐4H‐benzo[g]chromen‐2‐yl)benzyl)oxy)carbonyl) amino) ethyl methacrylate, O‐nitrobenzyl ether	[77]
		Mesoporous Prussian blue nanoparticles	[123]
	Ultrasound	Pluronic shells	[131]
	Magnetic	Magnetic iron oxide nanoparticles (IONPs)	[134]
H_2_S	No stimuli	Carboxyl‐functionalised mPEG‐PLGH‐thiobenzamide	[155]
	Light	Graphene oxide (rGO) nanosheets	[55]
	Intrinsic proteins	Poly (ethylene glycol)	[75]
	GSH	Lipids	[87]
	Magnetic	Lipids, superparamagnetic nanoparticles	[158]
SO_2_	GSH	mPEG‐PLG	[113]
	Light	Silica nanoparticles	[122]

## 5 CONCLUSION AND FUTURE PROSPECTS

GT is an emerging biomedical approach that requires peculiar focus on encapsulation, release and therapeutic action. A number of these nanoplatforms have been designed for treatment of cancerous, inflammatory, antibacterial and cardiovascular diseases. This approach is expected to gain more attention in the coming years, and it is prudent to gain some fundamental understanding by assessing recent progresses and challenges for this approach.

This review provides a concise discussion for responsive GRMs and the roles of nanomaterials utilised for carrying, delivering and facilitating gas release from GRMs. Many such responsible nanosystems will be discovered in the future, and current investigations should guide this quest to produce effective nanomedicines for GT.

As gases hold a lot of potentials for disease treatment in future clinic, researchers must ensure that their targeting is much more efficient because of the possibility of premature gas leakage, which may cause excessive biological toxicity that may also be witnessed in liquid or solid delivery systems. Therefore, it is highly required that gas‐releasing nanomedicines release gases only upon triggering by desirable intrinsic or extrinsic stimuli at specific target sites. Treatment with single gas releasing molecule may have some therapeutic effects, while combination of two or more gases and other therapeutic agents, coupled with multiple release strategies may ensure that GT is at an optimal level for clinical translation in future. Of course, to ensure that GT is suitable for clinical use, biocompatible delivery systems are desired for long term safety.

Currently, there are no approved gas‐releasing nanomedicines even though there are a few gas therapeutic platforms in clinical trials.^[^ ^159^ ^]^ Therefore, various other issues such as toxicity of residues of GRMs after gas release, off‐site targeting, and insufficient levels of gases released at diseased sites within the required period and potentially dangerous extrinsic stimuli must be resolved to steer these specific nanomedicines from bench to clinical testing and subsequent adoption for treatment of various diseases.

## CONFLICT OF INTEREST

Zhi Ping Xu is a member of the *Exploration* editorial board. The authors declare no conflict of interest.
